# Out-of-home food outlets and area deprivation: case study in Glasgow, UK

**DOI:** 10.1186/1479-5868-2-16

**Published:** 2005-10-25

**Authors:** Sally Macintyre, Laura McKay, Steven Cummins, Cate Burns

**Affiliations:** 1 Social & Public Health Sciences Unit, Medical Research Council, Glasgow, UK; 2 Department of Geography, Queen Mary, University of London, London, UK; 3 School of Exercise & Nutrition Science, Deakin University, Australia

## Abstract

**Background:**

There is a popular belief that out-of-home eating outlets, which typically serve energy dense food, may be more commonly found in more deprived areas and that this may contribute to higher rates of obesity and related diseases in such areas.

**Methods:**

We obtained a list of all 1301 out-of-home eating outlets in Glasgow, UK, in 2003 and mapped these at unit postcode level. We categorised them into quintiles of area deprivation using the 2004 Scottish Index of Multiple Deprivation and computed mean density of types of outlet (restaurants, fast food restaurants, cafes and takeaways), and all types combined, per 1000 population. We also estimated odds ratios for the presence of any outlets in small areas within the quintiles.

**Results:**

The density of outlets, and the likelihood of having any outlets, was highest in the second most affluent quintile (Q2) and lowest in the second most deprived quintile (Q4). Mean outlets per 1,000 were 4.02 in Q2, 1.20 in Q4 and 2.03 in Q5. With Q2 as the reference, Odds Ratios for having any outlets were 0.52 (CI 0.32–0.84) in Q1, 0.50 (CI 0.31 – 0.80) in Q4 and 0.61 (CI 0.38 – 0.98) in Q5. Outlets were located in the City Centre, West End, and along arterial roads.

**Conclusion:**

In Glasgow those living in poorer areas are not more likely to be exposed to out-of-home eating outlets in their neighbourhoods. Health improvement policies need to be based on empirical evidence about the location of fast food outlets in specific national and local contexts, rather than on popular 'factoids'.

## Background

Obesity is associated with a range of disorders including coronary heart disease, diabetes, kidney failure, osteoarthritis, cancer, back pain, and psychological damage [1]. Rates of overweight and obesity are high, and rising, in developed countries, and considerable concern has been expressed in a number of countries about this increase [2-4], described by the Chief Medical Officer for England as 'a ticking time bomb' [5].

The increasing prevalence of overweight and obesity has been linked to increasing physical inactivity and changes in eating patterns [6]. There has been an increase in the consumption of foods outside the home, and increases in portion size in out-of-home outlets (particularly 'fast food' outlets) [7-9]. In the UK in 2002–3, 27% of expenditure on food and drinks (excluding alcohol) was spent on consumption outside the home [10].

Foods eaten outside the home are often higher in energy and fat than foodstuffs prepared at home ; and it has been suggested that:

'the high energy densities of many fast foods challenge human appetite control systems with conditions for which they were not designed. Among regular consumers this is likely to result in the accidental consumption of excess energy and hence to promote weight gain and obesity' [7] p187.

Studies of fast food restaurant use have shown positive associations with intake of total energy and percent fat, and negative associations with intakes of fibre [11,12]. A longitudinal study found that people who ate meals from fast-food restaurants more than twice a week, at both baseline and 15 year follow up, gained 4.5 kilos more weight and had 104% greater increase in insulin resistance than people who ate less than one meal at a fast-food restaurant each week [13]. One study in the USA found, at the state level, a correlation between density measures of fast-food restaurants (per resident and per square mile) and obesity rates [14], although another study found that childhood overweight was not associated with proximity to fast-food restaurants in Cincinnati [15].

Dietary intake among poorer socio-economic groups is less likely to meet current nutritional guidelines for fruit and vegetable consumption [16], and more likely to be high in fat, salt and sugar (typically features of fast food) [17]. In the UK this applies to socio-economic status (SES) whether measured by household occupational social class or area-based measures of deprivation [18,19]. In developed countries obesity rates in women rise linearly with decreasing SES; the pattern for men is less straightforward and there are less steep, and in some places no or non linear, SES gradients in men [18-21]. However, area deprivation has consistently been shown to be related to overweight and obesity, even in models which take individual socio-demographic characteristics into account [22-24].

This observation has led to researchers to hypothesise that deprived areas may have fewer outlets selling healthy foods at affordable prices [25], but be better supplied by fast food outlets selling foods that are high fat and energy dense [26]. Evidence on the first point is equivocal, some studies finding poorer access in more deprived areas to supermarkets selling foods recommended in current dietary guidelines [27-30], but other studies finding either no socio-economic differences in shop locations or more supermarkets in poorer areas [31-37].

There is less available evidence on the second point. A study in Melbourne, Australia, found that there was a dose-response relation between SES and the density of fast food outlets, with people living in the poorest SES areas having 2.5 times greater exposure to fast food outlets than people in the wealthiest areas [26]. A study in New Orleans found that people living in low income and predominantly black areas had significantly more exposure to fast food restaurants [38]. Cummins et al found that in England and Scotland there was a higher density of McDonald's restaurants per thousand population in more deprived areas [39]. Although these are the only existing empirical studies on this issue, it is commonly suggested that residents of poorer neighbourhoods are more exposed to fast food outlets, e.g. in a frequently cited comprehensive review of obesity it is stated that:

'Poorer neighbourhoods tend to have fewer recreation amenities, be less safe, and have a higher concentration of fast food outlets' [20]p133.

Scotland has high rates of premature mortality which have often been attributed to poor diets, in particular high consumption of sugar, fried foods, and carbonated soft drinks [18,40]. Glasgow has a reputation for poor health and poor diet [41] and contains a higher proportion of deprived neighbourhoods than any other area in Scotland [42]. However, Glasgow has a considerable range of health and deprivation indices. We therefore considered it a good location for a case study which aimed to test the hypothesis that fast food outlets are more likely to be found in poorer neighbourhoods in the UK.

## Design and Methods

### Identification and classification of food outlets

We obtained lists of out-of-home food outlets in Glasgow in 2003 from the Food Safety Unit of Glasgow City Council. All food premises must be registered with the Council in accordance with the Food Safety (General Food Hygiene) Act 1995. A database was created containing all food outlets listed as restaurant, café or takeaway by the Council, and the full unit postcode of each outlet. This categorisation was mutually exclusive and based on the main activity of the outlet. However in practice these categories were not mutually exclusive; many restaurants or cafes (including Indian restaurants, fish and chip restaurants, burger restaurants, coffee shops and ice cream parlours) also provide a take away service. Restaurants include fine dining independent restaurants, vegetarian restaurants, ethnic restaurants, and fast food restaurants so their sales of high energy high fat foods may vary considerably. We separated restaurants into restaurants (independent and chain) or fast food chain. Chain fast food restaurants are national or international multi-outlet companies or franchises, whose outlets provide tables and chairs, but no crockery or cutlery, and counter service only; in Glasgow these include McDonalds, Burger King, KFC, Pizza Hut, and Wimpy. Many independent restaurants in Glasgow specialise in 'ethnic' food and are categorised in the local yellow pages as such (for example, Italian, Indian, Thai, Chinese etc). Many takeaways in Glasgow sell a range of foodstuffs (e.g. chips, pies, pizzas, curry, kebabs, burgers) all of which are typically energy dense.

### Classification of neighbourhood deprivation

Our primary measure of neighborhood deprivation was the Scottish Index of Multiple Deprivation (SIMD), created by the government for monitoring and planning purposes. The SIMD is calculated using data such as current income, employment, health, education, skills and training, telecommunications, and housing at the level of data zones [43]. Data zones are statistical areas which nest within local authority boundaries and are smaller than postcode sectors or wards. Data zones are intended to be effective in identifying small areas with particular social characteristics, and are therefore more internally homogeneous than postcode sectors. Look-up tables are available which relate individual post codes to data zones [44]. As the SIMD score increases the level of deprivation increases. We divided data zones into quintiles by SIMD scores (Quintile 1 covers 138 data zones, and quintiles 2–5 each cover 139). The mean population per data zone was highest (at 859.2) in quintile 1 (the least deprived) and lowest (at 815.1) in quintile 5 (the most deprived); the overall mean was 832.7 persons, standard deviation 152.6.

Initially we also used another area-based measure of deprivation, Carstairs scores, in addition to SIMD. This was because we were unsure about the appropriate spatial scale at which to measure access to out of home food outlets, and wished to check whether the spatial scales used made any difference to the analysis. Carstairs scores are based on the proportions of: overcrowded households, heads of household in social classes IV and V, unemployed male heads of household, and non-owner occupied properties, at the post code sector level using data in from the 2001 Census [42]. Post code sectors in Glasgow have a mean population size of 5556 and standard deviation of 3109. Four postcode sectors (G1 3, G2 2, G2 5, G2 6), containing 102 outlets (57 restaurants, 5 fast food chain restaurants, 21 cafes and 29 takeaways), do not have Carstairs scores, because of small populations. We found the results using Carstairs (results not shown, available from authors) followed a similar pattern to that using SIMD but we report the latter analysis only, as all food outlets and areas of the city were included, and data zones were smaller and less variable in size than post code sectors.

We are not assuming that people are restricted to food outlets within their own data zone. However, small areas in the UK are very clustered by deprivation (i.e. deprived areas tend to be adjacent to other deprived areas, and affluent areas to affluent areas) so if out of home outlets are concentrated in deprived areas these are also likely to effect the exposure of residents in adjacent, also deprived, areas.

### Analysis strategy

The mean number of eating outlets per 1000 persons was calculated using population data (2001 Census) for each data zone within each SIMD quintile. Statistical comparison between quintiles in outlet density was determined by ANOVA. Accepted level of significance was p < 0.05. More than half of the data zones had no outlets of any kind, so we also used Logistic Regression to determine the probability, in terms of Odds Ratios (OR) and Confidence Intervals (CI), of data zones within quintiles having any of the various outlets. Data zones grouped within Quintile 2 were used as the reference category since they contained most food outlets of all kinds. All analysis used SPSS Version 12.0 for Windows.

We also used MapInfo Professional Client 6.0 to map the different outlets on a base map of Glasgow to describe their distribution geographically to complement the statistical analysis by deprivation (map 1).

## Results

We identified 1301 out-of-home eating outlets in Glasgow; 339 restaurants, 30 fast food chain restaurants, 303 cafes and 629 takeaways. Thirty five per cent of these outlets, and nearly 50% of the fast food chain restaurants, were located in Q2, the second least deprived quintile (see table 1). The highest density per thousand population of each type of outlet, and for all combined, was in Q2 and the lowest density was in Q4. Differences between quintiles were statistically significant for restaurants and for takeaways.

**Table 1:** Mean number of food premises per 1000 people per SIMD Quintile

	**Restaurants**		**Fast food chains**		**Cafés**		**Takeaways**		**All outlets**	
	**Mean**	**N**	**Mean**	**N**	**Mean**	**N**	**Mean**	**N**	**Mean**	**N**
*SIMD Quintile (population)										
**1 Most Affluent **(118,568)	0.58	66	0.02	3	0.37	43	0.61	71	1.58	183
**2 **(114,744)	1.51	164	0.13	14	0.77	88	1.61	192	4.02	458
**3 Middling **(116,074)	0.48	58	0.05	6	0.61	70	1.36	149	2.51	283
**4 **(115,178)	0.12	15	0.00	0	0.26	29	0.82	92	1.20	136
**5 Most deprived **(113,305)	0.29	36	0.07	7	0.61	73	1.06	125	2.03	241
**Total **(577,869)	0.60	339	0.05	30	0.52	303	1.09	629	2.27	1301
**Sig (ANOVA)**	0.045		0.300		0.260		0.041		0.092	

*SIMD Quintile 1 includes 138 Data zones while Quintiles 2–5 include 139 Data zones each.

Table 2 outlines the results from the logistic regression analysis. Using Q2 as the reference category there was a statistically significant reduced probability of having any restaurants in data zones within Q3 (OR 0.45, 95% CI 0.24 to 0.82), Q4 (OR 0.21, 95% CI 0.10–0.43), and Q5 (OR 0.18, 95% CI 0.09–0.40). The probability of restaurants being present in data zones in Q1 was also lower but confidence intervals included 1 (OR 0.77, 95% CI 0.45–1.33). Confidence intervals were wide for fast food chain restaurants given their small number and estimates are thus unreliable. For cafes, the probability of data zones having any outlets was lower in each quintile than in the reference category, but only in Q4 did the confidence interval for the estimated odds ratio not include 1 (OR 0.40, 95% CI 0.22–0.73). Takeaways showed no clear pattern with the only statistically significant result being for Q1 (OR 0.49, 95% CI 0.29–0.81). Finally, combining all types of outlet, the probability of data zones having any outlet was lower in quintiles 1, 3, 4 and 5 than 2, though for Q3 the difference was non-significant (95% CI 0.56–1.42). There was no evidence of a linear relationship between any of the categories of outlet and deprivation. Overall there was no evidence that poorer areas in Glasgow were more likely to contain any out-of-home eating outlets.

**Table 2:** Probability of data zones within SIMD quintiles containing any outlets (in comparison to quintile 2); Odds Ratios (OR) and 95% Confidence intervals (CI). Significant Odds ratios (OR) in Italics.

	**Restaurants**		**Fast food chains**		**Cafés**		**Takeaways**		**All outlets**	
	**OR**	**95%CI**	**OR**	**95%CI**	**OR**	**95%CI**	**OR**	**95%CI**	**OR**	**95%CI**
**Quintile**										
**1 Affluent**	0.77	0.45–1.33	0.49	0.12–2.10	0.61	0.35–1.06	*0.49*	0.29–0.81	*0.52*	0.32–0.84
**2 **(reference)	1.00		1.00		1.00		1.00		1.00	
**3 Middling**	*0.45*	0.24–0.82	0.83	0.25–2.78	0.90	0.53–1.52	1.23	0.76–1.98	0.89	0.56–1.42
**4**	*0.21*	0.10–0.43	0.00	0.00-0.00	*0.40*	0.22–0.73	0.71	0.43–1.16	*0.50*	0.31–0.80
**5 Deprived**	*0.18*	0.09–0.40	0.66	0.18–2.38	0.77	0.46–1.32	0.78	0.48–1.27	*0.61*	0.38–0.98
**Overall sig.**		0.00		0.89		0.03		0.01		0.01

*SIMD Quintile 1 includes 138 Data zones while Quintiles 2–5 include 139 Data zones each.

The map displays the location of all outlets and shows that out-of-home food outlets tend to be located in the city centre (which is a retail centre, theatre/concert hall/cinema/club/pub area, Central Business District, and transport hub containing the main bus station and two train stations); along arterial highways (Great Western Road, Maryhill Road, London Road); and in cosmopolitan areas which are busy with workers during the day and have active night-time economies (e.g. Byres Road, Pollokshaws Road). From maps overlain with quintiles of area deprivation it appears that all types of outlet tend to be more common in City Centre and West End type areas, and to be less common in the peripheral deprived social housing areas such as Easterhouse, Castlemilk, Drumchapel, and Pollok, and in the most affluent housing areas in the City (maps not shown, available from authors).

**Figure 1:**
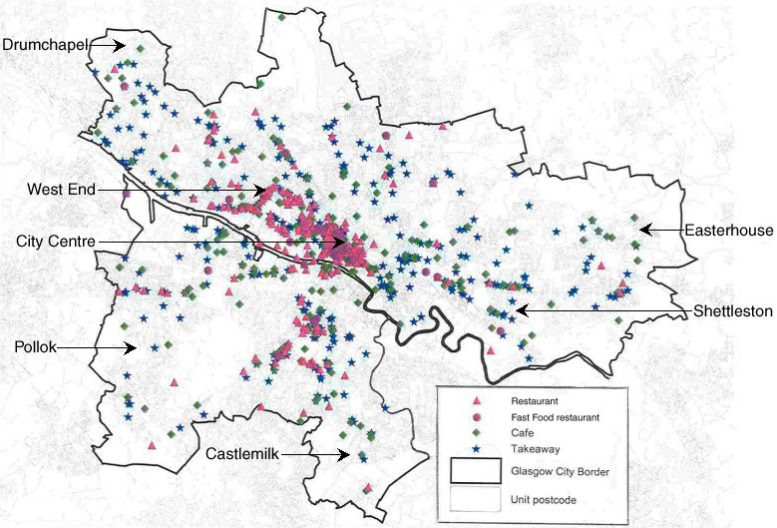
Map of Restaurants, Fast food Chain restaurants, Cafes and Takeaways in Glasgow City, 2003.

## Discussion

Whereas studies in Melbourne and New Orleans have found that fast food outlets were more concentrated in low income and black areas, we have found that neither out-of-home outlets in general, nor takeaways or fast food chain restaurants in particular, were more likely to be found in more deprived areas of Glasgow. To the contrary, we found that there were more outlets in the second most affluent category, and that outlets tended to be located in inner city or West End areas which attract customers during the day (e.g. retail and business centres) and evening (e.g. leisure centres). There are few outlets in areas which are primarily residential.

These findings may be unique to Glasgow and its particular history of urban development and planning, but we suspect the pattern we observed may be common in other UK cities. This is because restaurants and takeaways are likely to be located where there is most potential custom both during the day and evenings, and such demand is higher in retail, transport and commercial centres, areas with high density of entertainment facilities such as cinemas, theatres and pubs, and along arterial highways with much passing traffic. 'Gentrification' has involved the movement of socio-economically advantaged individuals into Glasgow City centre (e.g. 'the Merchant City') and the West End. Deprived areas in Glasgow are primarily residential and have often been noted to be lacking a whole range of local amenities (such as public transport, schools etc), especially the peripheral public housing estates (Castlemilk, Eaterhouse, Pollok, Drumchapel; see map) which were built immediately after the second world war to alleviate appalling housing conditions in the inner city. Thus the location of out-of-home eating outlets may reflect rational responses on the part of owners or franchisees to principles of supply and demand. This pattern may be different from those observed in the USA, where deprived populations may be concentrated more in the inner city and wealthier people dispersed to suburbs [34,45], and from Australia, where it has been argued by some that there is less spatial segregation along social and economic lines [46]. The different measures of deprivation used in the New Orleans, Melbourne, and Glasgow studies may also contribute to different findings.

Despite the fact that the location of out-of-home eating outlets may be a rational response to likely demand, there seems to be a prevailing assumption that such outlets, particularly fast food outlets, are targeted at deprived communities and are part of the reason for poorer diets and higher obesity levels in poor places. In an article in The Observer, a national Sunday newspaper, David Smith wrote about Shettleston, a relatively deprived area in Glasgow, which has one the worst life expectancy records in the UK. He gave considerable emphasis to the density of fast food outlets:

'At the front of Celtic FC's Parkhead Stadium, children queue for a burger bar at 10 in the morning....(and) Bridgeton, within the Shettleston constituency, is possibly the alcohol and fast food capital of Britain. Within a radius of just 200 yards around the metro station there are nine pubs, an off licence and seven takeaways'

and he quotes several local people similarly emphasising fast food availability:

"The children go to Burger Kings or McDonalds, and there's nothing you can do"...."There is a fast food shop at every corner. Going to those places becomes a habit" [47].....

While such stereotypes are common, they are not supported by the findings of this study. Though there are a number of out-of-home outlets along Shettleston Road and London Road (see map) their presence there is no more dense than along other main roads including ones in more middle class areas such as the West End. Just as perceptions on the part of the mass media, policymakers and food activists to the effect that deprived communities have poorer access to healthy foods at affordable prices may not be borne out by the empirical evidence, [34] perceptions held about a greater exposure among deprived communities to unhealthy diets may not necessarily be empirically substantiated.

There are clearly some limitations to our study. It is restricted to one city (as are those undertaken in Melbourne and New Orleans). By definition, we have not been able to map the location of mobile fast food outlets (e.g. vans selling pies, burgers etc) and it may be that these target poorer residential areas. It should also be noted that although takeaways and the chain restaurants primarily serve what is conventionally called fast food (i.e. high in fat, energy, and salt), they, and other restaurants and cafes, may serve healthier options instead of, or as well as, fast foods. Our interpretation of the maps is descriptive rather than using a more sophisticated GIS approaches.

Our findings may not appear consistent with those from another study in which we assessed the location of McDonald's restaurants in England and Scotland in relation to deprivation in 2005, and found that these restaurants were significantly more likely to be found in more deprived neighbourhoods[39]. Large global chains such as McDonald's have sophisticated marketing systems that allow them to pin-point with some accuracy the 'optimum' location based on geo-demographics, distance from headquarters, distribution points and main transport links and intersections, and sales figures in existing outlets [9,48] whereas the locational strategies of independent outlets may be more locally based. It may also be that individual chains avoid areas where others are located, so that if one chain tends to be located in more deprived neighbourhoods, others will locate in less deprived ones and so balance each other out.

The association between area level social deprivation and the availability of foods prepared outside the home, in particular fast foods, must be investigated in the context of the local availability of land, the price of real estate and the ease of obtaining planning permission. Differences in these factors may explain the differences between the findings reported here and those reported nationally for a single large global chain, and in other countries.

With increasing evidence of the possible risks to health of some types of fast food (because of portion sizes, energy density, and fat and salt content), it is important to establish whether the popular assumption that proximity to fast food outlets tends to lead to greater consumption of such foods and subsequently higher rates of obesity and poor health is substantiated. Further critical evaluation of the role of access to foods eaten outside the home in the aetiology of obesity is warranted. This relationship may well be more complex than simple proximity to an outlet, and may vary with macro and more local cultural and socioeconomic factors. As we have previously argued [49], it is important that health promotion policies in relation to the predicted obesity epidemic are based on robust empirical evidence and sensitivity to cultural and socioeconomic context, rather than on untested assumptions or 'factoids'.
